# Dietary Prebiotics, Milk Fat Globule Membrane, and Lactoferrin Affects Structural Neurodevelopment in the Young Piglet

**DOI:** 10.3389/fped.2016.00004

**Published:** 2016-02-04

**Authors:** Austin T. Mudd, Lindsey S. Alexander, Kirsten Berding, Rosaline V. Waworuntu, Brian M. Berg, Sharon M. Donovan, Ryan N. Dilger

**Affiliations:** ^1^Piglet Nutrition and Cognition Laboratory, Department of Animal Sciences, University of Illinois, Urbana, IL, USA; ^2^Neuroscience Program, University of Illinois, Urbana, IL, USA; ^3^Division of Nutritional Sciences, University of Illinois, Urbana, IL, USA; ^4^Department of Food Science and Human Nutrition, University of Illinois, Urbana, IL, USA; ^5^Mead Johnson Pediatric Nutrition Institute, Evansville, IN, USA

**Keywords:** prebiotics, milk fat globule membrane, lactoferrin, internal capsule, pig, nutrition, brain, neurodevelopment

## Abstract

**Introduction:**

Milk fat globule membrane (MFGM) and lactoferrin have been identified as two components that have potential to affect neurodevelopment. While concentrations of some MFGM constituents in infant formulas are within human milk range, they may not be present at optimal or clinically effective levels. However, lactoferrin levels of infant formulas are consistently reported to be lower than human milk. This study sought to provide a novel combination of prebiotics, bovine-derived MFGM, and lactoferrin and assess their influence on neurodevelopment.

**Methods:**

Twenty-four male piglets were provided either TEST (*n* = 12) or CONT (*n* = 12) diet from 2 to 31 days of age. Piglets underwent spatial T-maze assessment starting at 17 days of age, were subjected to magnetic resonance imaging at 30 days of age, and were euthanized for tissue collection at 31 days of age.

**Results:**

Diffusion tensor imaging revealed differences in radial (*P* = 0.032) and mean (*P* = 0.028) diffusivities in the internal capsule, where CONT piglets had higher rates of diffusion compared with TEST piglets. Voxel-based morphometry indicated larger (*P* < 0.05) differences in cortical gray and white matter concentrations, with CONT piglets having larger tissue clusters in these regions compared with TEST piglets. In the spatial T-maze assessment, CONT piglets exhibited shorter latency to choice compared with TEST piglets on day 2 of acquisition and days 3 and 4 of reversal.

**Conclusion:**

Observed differences in microstructure maturation of the internal capsule and cortical tissue concentrations suggest that piglets provided TEST diet were more advanced developmentally than piglets provided CONT diet. Therefore, supplementation of infant formula with prebiotics, MFGM, and lactoferrin may support neurodevelopment in human infants.

## Introduction

Early life is a period of rapid neurodevelopment and nutrition during this critical phase can have lasting effects on structural and functional neurodevelopment. Human milk is generally considered the optimal source of nutrient provision for the human infant, and some studies have demonstrated improved cognitive development of breastfed infants (1). Yet breastfeeding is not always a viable option, and it has been recognized that infant formula can substitute for human milk as the sole source of nutrition for infants. Compositional differences between human milk and infant formulas exist, thus creating a need to better understand whether specific components in human milk may influence a variety of developmental effects including postnatal brain development. Analysis of human and bovine milk revealed lactoferrin (Lf) and milk fat globule membrane (MFGM) as two key components produced throughout lactation (2, 3). Importantly, both Lf and MFGM are comprised of individual compounds that may independently exert beneficial effects on the developing brain, further justifying a need to elucidate their combined actions in early development.

Lactoferrin is an abundant functional protein in the whey fraction of human milk, which exerts antimicrobial effects and modulates immune responses (4–6). However, recent evidence suggests that this sialic acid-rich, iron-binding glycoprotein may also play an important role in neurodevelopment (7). Piglets supplemented with 0.6 g/L bovine Lf exhibited increased mRNA and protein concentrations of brain-derived neurotrophic factor (BDNF), phosphorylated cAMP response element-binding protein (pCREB), and polysialic acid in the hippocampus, all of which are expressed during learning events and memory consolidation (7). Moreover, dietary provision of Lf enhanced the cognitive function of piglets as assessed by an 8-arm radial maze. Furthermore, supplementation of Lf in piglets increased gene expression of BDNF and glial cell line-derived neurotrophic factor in the duodenum, suggesting its beneficial effects on the enteric nervous system (8). Lactoferrin concentration in cow’s milk (0.03–0.1 g/L) differs substantially when compared with human milk (0.44–4.4 g/L), thus raising the question of whether Lf should be supplemented in infant formulas (2).

Historically, MFGM has been discarded in the preparation of infant formulas, although many individual components of this membrane may positively impact neurodevelopment. The MFGM is composed of sialic acid, gangliosides, sphingomyelin, choline, glycerophospholipids, proteins, and cholesterol (9). Although these components are present in infant formula, their concentrations may fall at the low end of what is commonly observed in human milk (10). Infants supplemented with complex milk lipids exhibited increased serum ganglioside concentration and enhanced cognitive development when compared with control formula-fed infants (11). Sialic acid contained within the gangliosides may facilitate synapse formation, thereby resulting in the observed enhancement in cognitive development in the supplemented infants. Supplementation of sphingomyelin-fortified milk in preterm infants elicited shortened latency in visual-evoked potentials (VEP), higher novelty preference, and increased Colombo attention scores compared with control preterm infants (12). Moreover, infants provided a low-energy, low-protein formula supplemented with MFGM exhibited higher cognitive scores on the Bayley Scales of Infant and Toddler development compared with control-fed infants and were not different than breast-fed controls (9). Studies suggest that these individual components are important for neurodevelopment (9). Therefore, evidence suggests that supplementation of MFGM may enhance structural and functional neurodevelopment, yet knowledge of its underlying mechanisms appears to be lacking.

Emerging evidence suggests that dietary manipulation of the intestinal microbiome may impact the development and functions of the enteric and central nervous systems. Previous studies in humans, piglets, and rodents have shown that polydextrose and galactooligosaccharides can significantly alter the microbiome (13–15) and potentially the gut-brain axis by reducing visceral pain sensitivity caused by immune challenge (16). While such mechanisms may partially contribute to the brain related effects reported here, the microbiome and gut–brain axis related outcomes from this study are presented elsewhere (Berding et al., under review)^1^.

To our knowledge, no study has investigated a diet containing prebiotics, Lf, and MFGM and assessed their combined impact on whole brain development. Furthermore, research has demonstrated the omega-3 fatty acids docosohexaenoic acid (DHA) and arachidonic acid (ARA) are needed to support brain and visual development and as such are included in the vast majority of infant formulas on the market today. Importantly, this study was designed to seek additional brain benefits beyond DHA and ARA by including these in both the control and experimental formulations. Therefore, the aim of this study was to use the piglet as a pre-clinical model to elucidate potential mechanisms whereby a novel combination of prebiotics, Lf, and MFGM affected neurodevelopment beyond what is provided by DHA and ARA. We hypothesized that supplementation of this novel combination of ingredients would enhance the overall brain development of the supplemented group compared with piglets fed control formula.

## Materials and Methods

### Animals and Housing

All animal care and experimental procedures were in accordance with National Research Council Guide for the Care and Use of Laboratory Animals and approved by the University of Illinois at Urbana-Champaign Institutional Animal Care and Use Committee. Twenty-four naturally farrowed intact male Yorkshire piglets from the University of Illinois Imported Swine Research Laboratory were obtained 48 h after birth, to allow colostrum consumption, and artificially reared over a 30-day trial period. The trial was completed in two replicates (12 piglets per replicate), with four piglets selected from six litters to control for genetics and initial body weight. Piglets were individually housed, as previously described, in stainless steel cages (1.03 m deep × 0.77 m wide × 0.81 m high) with clear, Plexiglas facades and side walls bearing several small openings (2.54 cm in diameter) to allow for adequate ventilation. A towel and toy were included in each cage to provide enrichment, and piglets were allowed *ad libitum* access to water (17, 18).

Ambient room temperature was maintained between 27 and 29°C and heat lamps and mats provided supplemental heat within the cage. A 12 h light/dark cycle was maintained with light from 0600 to 1800 hours. Prior to placement in the artificial rearing system, piglets were administered 5 mL of *Clostridium perfringens* antitoxin C + D per the manufacturer’s recommendations (Colorado Serum Company, Denver, CO, USA). At 31 days of age, piglets were anesthetized using a telazol:ketamine:xylazine solution [50.0 mg tiletamine plus 50.0 mg of zolazepam reconstituted with 2.50 mL ketamine (100 g/L) and 2.50 mL xylazine (100 g/L); Fort Dodge Animal Health] by intramuscular injection at 0.03 mL/kg BW. After verifying anesthetic induction, piglets were euthanized via intracardiac administration of sodium pentobarbital (86.0 mg/kg of body weight; Fatal Plus, Vortech Pharmaceuticals, Dearborn, MI, USA) to permit collection of tissue samples.

### Dietary Treatments

All researchers involved with conducting the study and acquiring and analyzing study results remained blinded to dietary treatment identity until final data analyses had been completed. Piglets (*n* = 12 per diet) were provided either a control (CONT) or test (TEST) diet, which was supplemented with the following (g/100 g milk replacer powder): prebiotic blend of polydextrose/galactooligosaccharides (PDX/GOS) (1.2 g/100 g PDX, Danisco, Terre Haute, IN, USA; 3.5 g/100 g GOS, FrieslandCampina, Zwolle, Netherlands), Lf (90.4% pure, 11% iron saturation; 0.3 g/100 g; Tatua Cooperative Dairy Company, Morrinsville, New Zealand), and MFGM-10 (2.5 g/100 g; Arla Food Ingredients, Aarhus, Denmark) (Mead Johnson Pediatric Nutrition Institute, Evansville, IN, USA). Both CONT and TEST diets were supplemented with docosahexaenoic acid (91 mg/100 g milk replacer powder) and ARA (182 mg/100 g milk replacer powder). In this study PDX/GOS was provided for its prebiotic effects on gut microbiota. As such, data regarding the effects of PDX/GOS on microbiome composition can be found in the supplemental manuscript and were not included in the analyses contained herein (Berding et al., under review)^1^.

Both CONT and TEST milk replacer powder was reconstituted at 200 g of dry powder per 800 g of water. At this reconstitution rate, both diets contained docosahexaenoic acid (182 mg/L) and ARA (364 mg/L), and the reconstituted TEST treatment contained PDX/GOS (2.4 and 7 g/L of PDX and GOS, respectively), Lf (0.6 g/L), and MFGM (5.0 g/L). Piglets received small volumes of milk treatments on the day of arrival to provide an adjustment period prior to the standard feeding regimen. Piglets were fed at 285, 305, and 310 mL of reconstituted diet per kg BW starting on 3, 5, and 12 days of age, respectively. Body weight was recorded daily to determine the volume of milk to be dispensed to individual animals throughout the day. Meals were administered five times a day, approximately every 4 h, between 0700 and 2200 hours, and each diet was reconstituted fresh at each feeding. Piglets were fasted prior to cognitive testing to incentivize the milk reward offered in the behavioral task. On days that behavioral testing occurred (between 17 and 28 days of age) piglets were provided four meals instead of five, while maintaining the aforementioned daily volume per kg body weight feeding rate.

### Behavioral Testing

Hippocampal-dependent learning and memory was assessed using a validated behavioral task of spatial working memory in a specially designed T-maze (19). All researchers involved in the behavioral assessment were blinded to dietary treatment throughout behavioral testing. Starting at approximately 1 week of age piglets were individually socialized and allowed to explore the rearing room for approximately 5 min each morning. During this time researchers interacted with piglets and observed piglet health and signs of lameness. Beginning at 17 days of age, piglets were subjected to 11 days of behavioral testing consisting of a 7-day acquisition phase followed by a 4-day reversal phase. Following an overnight fast to ensure adequate motivation for a food reward, piglet were tasked with locating 5 mL of milk replacer from the designated dietary treatment in a constant direction (either north or south) within 60 s. Piglets were randomly started on either the east or west ends of the maze to ensure the use of extra-maze visual cues and not an egocentric mechanism to solve the task. Following acquisition, piglets were transitioned to a reversal phase, in which the reward direction was converse to that used during the acquisition phase, compelling the piglets to learn the new location of the reward.

Piglets underwent a series of 10 trials per day with the number of correct choices as a proportion of 10 total choices used as the primary response parameter, and incidence of non-compliance (i.e., proportion of total choices where pig did not complete the task within 60 s) was also recorded. Latency to choice, or the number of seconds to complete the task, was determined manually by stopwatch in the first replicate and a time stamp hard-coded in a recorded video was used for the second replicate. Because the methodology differed between the two replicates, the time per trial for each pig was rounded to the whole second.

Individual piglet performance was evaluated in three stages to determine task participation before further analysis of learning was measured. The first phase documented the frequency of non-compliance of each trial for individual pigs. Next, participation was determined by the number of occurrences of non-compliance. Piglets were allowed up to three non-compliant trials per day before being considered non-participatory, and the entire day marked as non-compliant. Finally, if three or more days of non-compliance were documented for an individual pig, that subject’s behavioral data were removed from the final dataset.

### Magnetic Resonance Imaging

At 30 days of age, piglets underwent magnetic resonance imaging (MRI) procedures. Piglets were scanned on a Siemens MAGNETOM Trio 3T Imager using a Siemens 32-channel head coil. Upon arrival to the Beckman Institute Biomedical Imaging Center, piglets were anesthetized via intramuscular injection of Telazol (0.07 mg/kg body weight; Zoetis, Florham Park, NJ, USA). Once sedated, piglets were transferred to the MRI scanner and maintained on 2% isoflurane/98% oxygen for the entirety of the 60 min scan. An MRI-compatible pulse oximeter was used to monitor piglet vital signs throughout the scan. Upon completion of the scan, vital signs were monitored and recorded until complete recovery from anesthesia. Specific details regarding piglet imaging sequences and post-imaging analysis of diffusion tensor imaging (DTI) and voxel-based morphometry were previously described (18, 20).

### Manual Brain Segmentation

Scanning procedures provided three 3D T1-weighted magnetization-prepared rapid gradient echo (MPRAGE) scans per piglet, with a 0.7 isotropic voxel size. Procedures for MPRAGE averaging and manual brain extraction were previously described (18). *A priori* tissue probability mapping and assessment of 19 different brain regions was performed using the Piglet Brain Atlas, validated previously, and publicly available at http://pigmri.illinois.edu (21).

### Volumetric Assessment

Individual brains were segmented into 19 different regions of interest (ROI) using the piglet brain atlas. Total brain and individual region volume analysis was performed on diffeomorphic anatomical registration using exponentiated Lie algebra (DARTEL)-generated warp files for each region using the fslstats toolbox provided in the FSL 5.0 package (Analysis Group, FMRIB, Oxford, UK). Generation of region-specific warp files was previously described (20).

### Voxel-Based Morphometry

Voxel-based morphometry (VBM) analysis was performed, to assess gray matter and white matter tissue concentrations using SPM8 software (Wellcome Department of Clinical Neurology, London, UK). Manually extracted brains were aligned to piglet brain atlas space using a 12-parameter affine transformation. The “Segment” function of SMP and piglet-specific prior probability tissue maps were then used to segment the brains into gray matter, white matter, and cerebrospinal fluid. The DARTEL toolbox was used with the same piglet-specific specifications as described previously (20).

### Diffusion Tensor Imaging

Diffusion tensor imaging values were generated using a diffusion-weighted echo-planar imaging sequence with a *b*-value of 1000 s/mm^2^ and 30 directions. Fractional anisotropy (FA), axial diffusivity (AD), radial diffusivity (RD), and mean diffusivity (MD) values were generated for cortical white matter, caudate, corpus callosum, cerebellum, internal capsule, thalamus, and both hippocampi using methods previously described (18).

### mRNA Expression

Ribonucleic acid was extracted from the right hippocampus and prefrontal cortex brain tissues using RNeasy Plus Mini Kit (QIAGEN, Venlo, Limburg) according to manufacturer’s instructions. Extracted RNA yield was quantified by spectrophotometry (NanoDrop 1000; Thermo Fisher Scientific, Waltham, MA, USA) at 260 nm and quality was assessed with the 2100 Bioanalyzer (Agilent Technologies, Santa Clara, CA, USA) in the W.M. Keck Center at the University of Illinois; all samples had a RIN > 6. Reverse transcription was performed using 2 μg of total RNA in a volume of 10 μL using the High Capacity cDNA Reverse Transcription kit (Life technologies, Carlsbad, CA, USA).

Quantitative real-time PCR was conducted for BDNF (TaqMan Expression Assay: Ss03822335_s1, Life Technologies) in both right hippocampus and prefrontal cortex samples on a MicroAmp optical 384-well plate (Life Technologies, Carlsbad, CA, USA) using the 7900HT Fast Real-Time PCR system (Life Technologies, Carlsbad, CA, USA). Ribosomal protein L19 (RPL 19; Ss03375624_g1) was used as an endogenous control. Sample mRNA abundance was quantified with the use of the Relative Standard Curve method, where the standard curve, derived from a stock of pooled porcine hippocampal and midbrain cDNA, was prepared using serial 1:5 dilutions. Normalized values were calculated by dividing the target quantity mean by the RPL19 quantity mean, while fold-change differences were calculated by dividing the normalized target value (TEST) by the average normalized calibrator (CONT) value.

### Statistical Analysis

An analysis of variance (ANOVA) was conducted using the MIXED procedure of SAS 9.3 (SAS Inst. Inc., Cary, NC, USA) was applied to differentiate the effects of the CONT vs TEST diets provided to young pigs. Depending on the outcome, one of two statistical models used was as follows: (1) any data collected at a single time-point (i.e., brain volume, DTI, fatty acid, and gene expression) was analyzed by a simple one-way ANOVA and (2) any data collected from the same animal on more than one occasion (i.e., behavioral outcomes) were analyzed as a two-way, repeated-measures ANOVA. Both statistical models included replicate as a random effect and the level of significance was set at *P* < 0.05 with trends accepted at 0.05 < *P* < 0.10.

### Voxel-Based Morphometry Statistical Analysis

Statistical analysis of VBM outcomes was performed as previously described (20). Due to the small sample size of this study, the statistical non-parametric (SnPM13) methods toolbox was used for proper analysis (http://warwick.ac.uk/snpm). As part of this analysis, non-parametric permutation and randomization tests were performed, followed by a two-sample *t*-test to compare CONT vs TEST diets. This analysis did not include any covariates and an analysis of covariance (ANCOVA) was used for global normalization. Statistical maps using pseudo-*t* values were generated to show regional differences in gray and white matter between the two treatment groups. An uncorrected alpha of 0.01 was used to ensure proper statistical significance for individual comparisons. An additional threshold of at least 20 edge-connected voxels was used to count a voxel cluster as significant.

## Results

### Piglet Growth and Health

Piglets in this study grew at rates consistent with artificially and sow-reared piglets of similar age. No signs of lameness or sickness were observed during daily observations of piglets on this study. For more informative data regarding piglet growth and health, the reader is referred to our companion paper (Berding et al., under review)^1^.

### Behavioral Assessment

**Figure 1:**
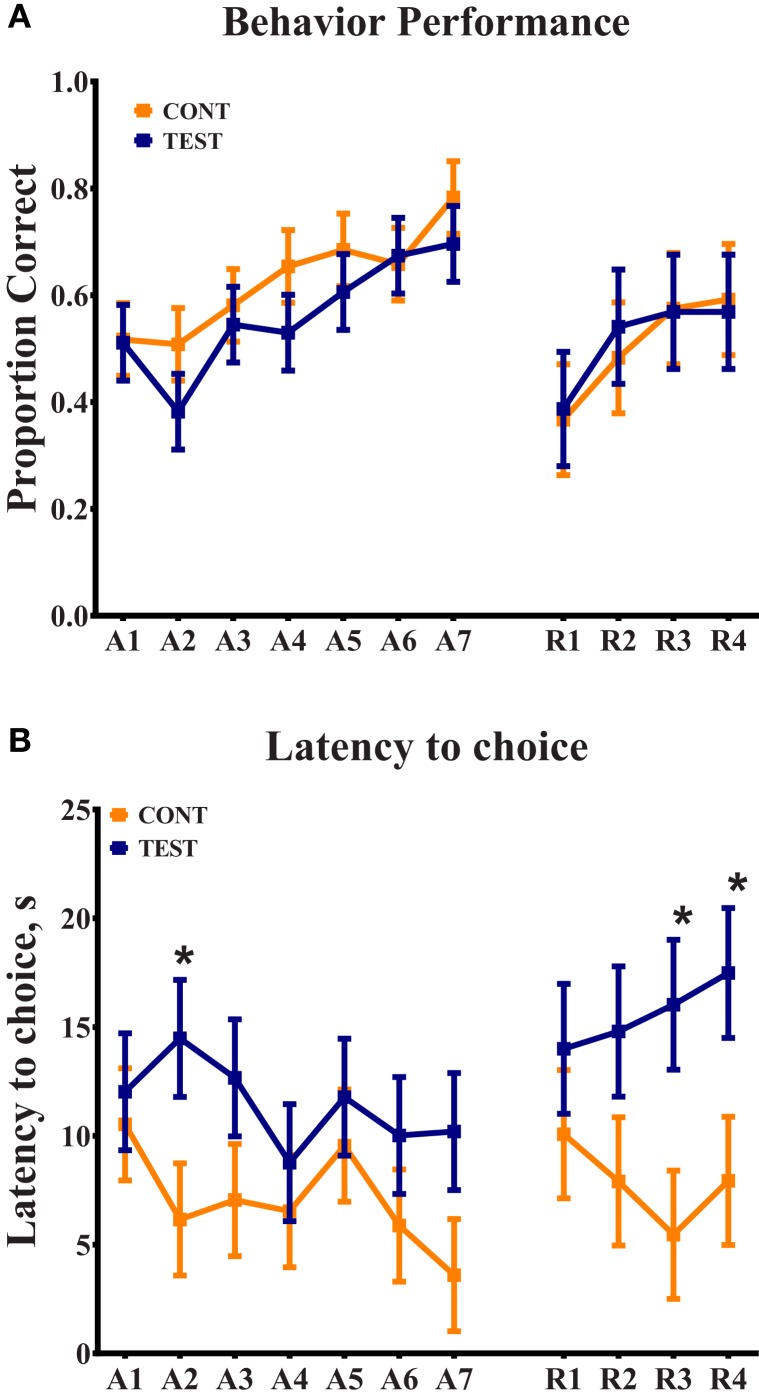
**Spatial T-maze behavioral assessment revealed differences due to diet in latency to choice, on three separate days**. **(A)** Behavior performance was assessed by proportion of correct choices, out of 10 trials, per day. No differences were observed between dietary treatments. **(B)** Analysis of latency to choice revealed differences (*P* < 0.05) on day 2 of acquisition and days 3 and 4 of reversal, in all cases TEST piglets exhibited longer latencies compared with CONT piglets on these days.

Based on the participation criteria set forth in this study, one piglet from the TEST diet was excluded from the behavioral dataset due to non-compliance on third day of the 11-day assessment period. Thus, 11 piglets from the TEST diet and 12 piglets from the CONT diet were included in the final dataset. No interactive effects (i.e., diet × day interaction) or the main effect of diet was observed for percentage of correct choices throughout the 11-day assessment (Figure 1); only a main effect of day (*P* < 0.05) was evident. When analyzing latency to choice, no interactive effect was noted, but there was a trend (*P* = 0.079) for diet to influence this outcome. Thus, piglets provided the TEST diet took longer to make a choice on day 2 (*P* = 0.027) of the acquisition phase and days 3 (*P* = 0.012) and 4 (*P* = 0.023) of the reversal phase, when compared with CONT piglets.

### Brain Volume Analysis

Due to excessive motion during MRI data acquisition, one piglet from the TEST diet was excluded from all MRI analyses. Thus, 11 piglets from the TEST diet and 12 piglets from the CONT diet were used. Absolute brain volumes were not different between dietary treatment groups. When brain regions were assessed relative to total brain volume within subject, there were no differences between diets observed in relative volume for any of the 19 anatomical regions analyzed. Analysis of brain volumes relative to intracranial volume (i.e., total brain volume) is consistent with current neuroimaging practices.

### Voxel-Based Morphometry

**Figure 2:**
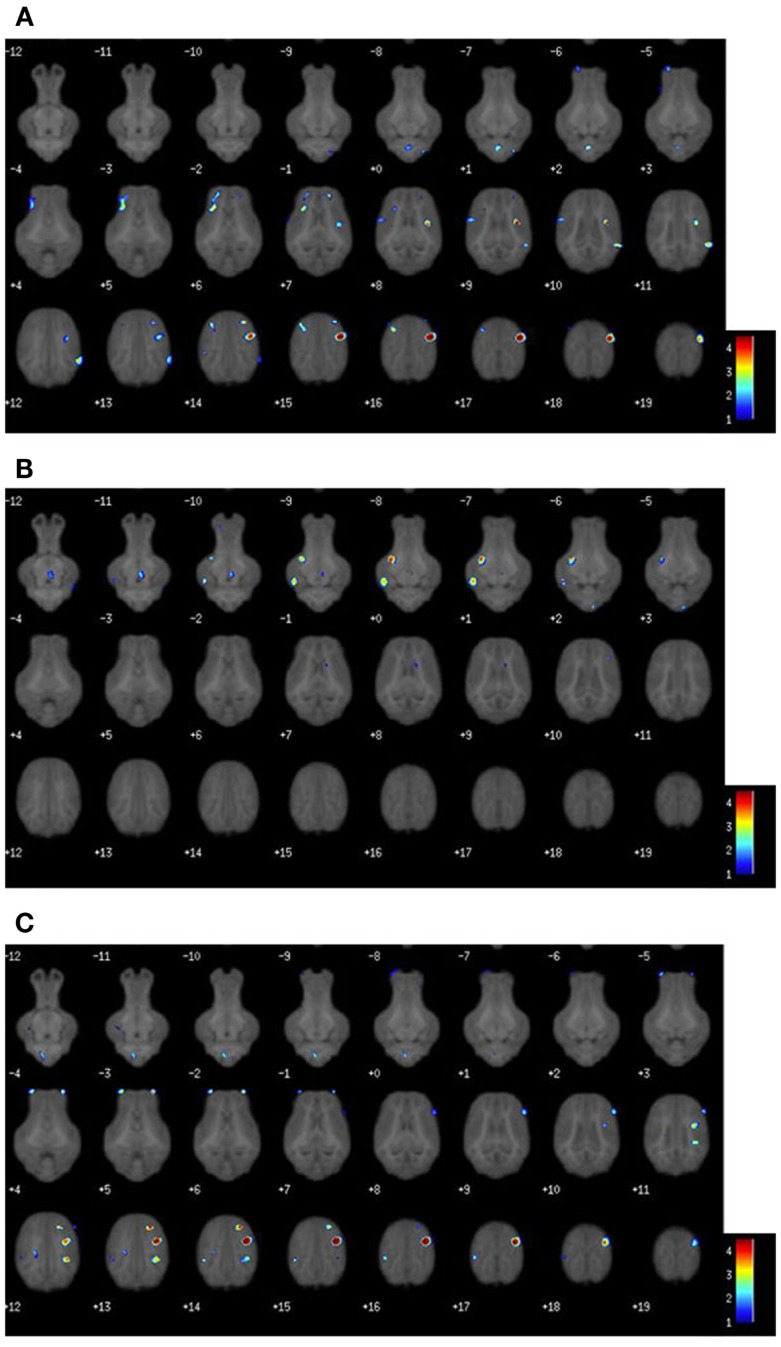
**Voxel-based morphometry heat maps illustrating tissue concentration differences between TEST- and CONT-fed piglets**. The color bar indicates pseudo-*t* statistics, used to determine the *P*-uncorrected statistics listed in Table 1. **(A)** Gray matter tissue differences in which CONT piglets have more gray matter than TEST piglets. **(B)** Gray matter tissue differences in which TEST piglets have more gray matter than CONT piglets. **(C)** White matter tissue differences in which CONT piglets have more white matter than TEST piglets. Illustration for white matter differences where TEST piglets have more white matter than CONT, not shown.

Voxel-based morphometric analysis of gray and white matter tissue segmentations revealed differences in regional tissue concentrations between treatment groups (Table 1). A comparison of gray matter concentration in which CONT piglets had higher (*P* < 0.05) regional peak intensities of gray matter compared with TEST piglets (CONT > TEST) revealed specific differences in cerebellum, left cortex, and right cortex (Figure 2). When analyzing regional clusters in which gray matter in TEST piglets was more concentrated than in CONT piglets (TEST > CONT), significant peak intensities in the left cortex were again evident, although was a smaller number of significant voxels. White matter concentration in which CONT > TEST resulted in large regional clusters within the right cortex, left cortex, and medulla. Only small voxel cluster differences in white matter concentration were observed when TEST > CONT comparisons were analyzed, with the largest cluster being centered in the right cortex.

**Table 1:** **Voxel-based morphometry^a^**.

Tissue	Comparison	Anatomic region^b^	Cluster	Peak level		Local maxima coordinates^c^		
		
			(# voxels)	*P*-value	Pseudo-*t*	*x*	*y*	*z*
Gray	CONT > TEST	Cerebellum	147	0.004	3.40	0	−18	2
		Cerebellum	41	0.010	2.89	10	−19	1
		Left cortex	547	0.006	3.41	−10	20	6
		Left cortex	173	0.010	3.06	−11	20	16
		Left cortex	149	0.005	2.44	−18	13	10
		Left cortex	129	0.008	2.27	−6	36	3
		Right cortex	1051	< 0.001	6.28	15	13	16
		Right cortex	371	0.009	3.81	20	−3	10
		Right cortex	86	0.003	3.29	11	23	14
		Right cortex	42	0.007	2.82	8	29	7
	TEST > CONT	Caudate	40	0.006	1.56	6	15	8
		Cerebellum	26	0.009	3.01	3	−24	3
		Cerebral aqueduct	26	0.010	2.50	1	−2	−2
		Left cortex	392	0.005	4.12	−11	9	1
		Left cortex	299	0.010	3.96	−17	−6	2
		Midbrain	34	0.010	2.13	−6	−6	−8
		Right cortex	37	0.009	1.77	17	−9	−4
White	CONT > TEST	Cerebellum	254	0.009	3.61	−1	−20	−1
		Left cortex	143	0.009	3.62	−15	1	15
		Left cortex	480	0.007	3.45	−10	35	5
		Left cortex	62	0.010	2.80	−9	6	13
		Left Hippocampus	100	0.009	1.25	−12	−2	−4
		Medulla	712	0.006	3.66	−6	−15	−14
		Right cortex	1026	0.001	7.10	14	13	15
		Right cortex	151	0.001	4.57	9	22	13
		Right cortex	175	0.006	4.12	10	35	5
		Right cortex	183	0.009	4.02	15	1	12
		Right cortex	70	0.008	1.23	9	27	0
	TEST > CONT	Cerebellum	29	0.009	1.88	−4	−16	3
		Lateral Ventricle	21	0.010	2.18	4	20	8
		Right cortex	203	0.009	1.19	18	−8	6

*^a^Voxel-based morphometry analysis of gray and white matter differences in the TEST and CONT piglet brains. A threshold of *P* < 0.01 and minimum cluster size of 20 voxels were used to determine *P*-uncorrected values listed in the table*.

*^b^Brain regions based on estimates from the University of Illinois Piglet Brain Atlas (http://pigmri.illinois.edu)*.

*^c^Local maxima coordinates: *x* increases from left (−) to right (+), *y* increases from posterior (−) to anterior (+), and *z* increases from inferior (−) to superior (+)*.

### Diffusion Tensor Imaging

**Figure 3:**
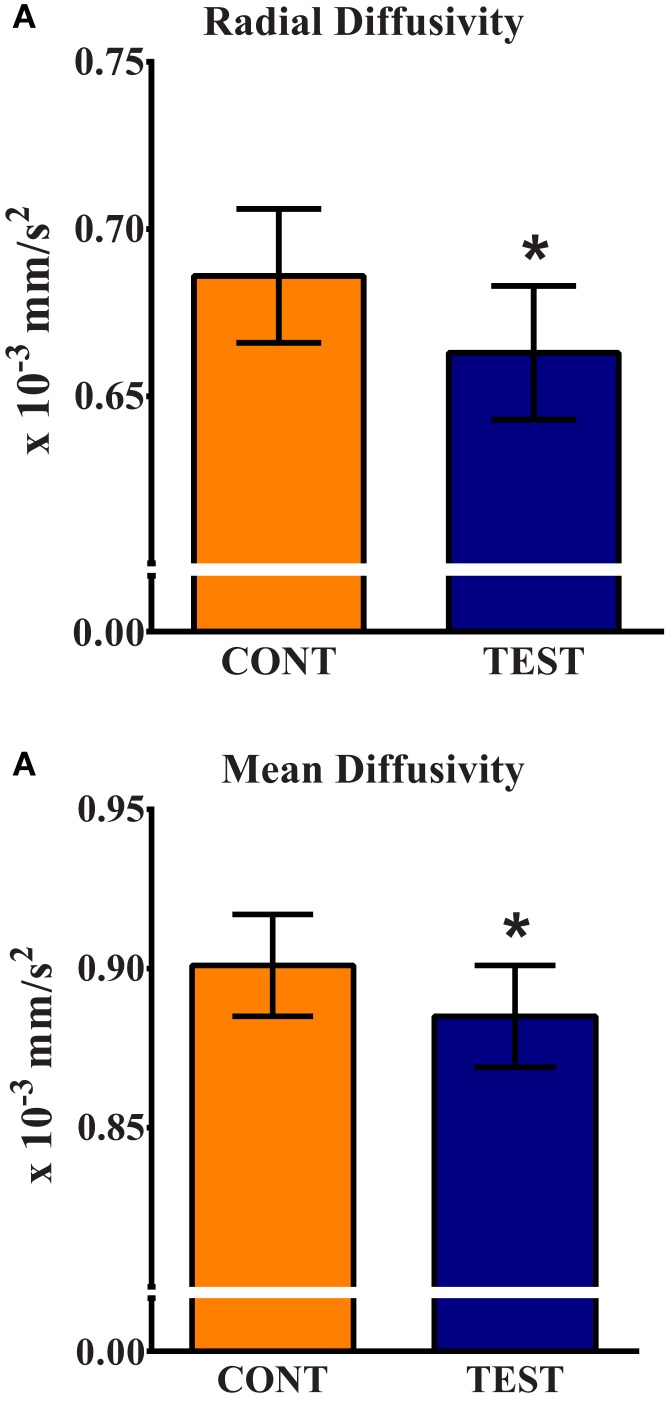
**Radial and mean diffusivity indicate greater maturation in the internal capsule of TEST-fed piglets compared with CONT**. **(A)** Radial diffusivity measures revealed CONT piglets had higher (*P* = 0.032) rates of diffusion compared with TEST piglets. **(B)** Mean diffusivity measures revealed CONT piglets had higher (*P* = 0.028) rates of diffusion compared with TEST piglets. * denotes significant (*P* < 0.05) difference between TEST and CONT piglets.

Assessment of water molecule diffusion revealed differences (*P* < 0.05) due to dietary treatment in radial and MD measures within the internal capsule. RD measures in the internal capsule indicated higher (*P* = 0.032) rates of diffusion in the CONT piglets compared with the TEST piglets (Figure 3). MD in the internal capsule denoted higher (*P* = 0.028) rates of diffusion in the CONT piglets compared with the TEST piglets. No differences were observed in any other brain region for RD, MD, AD, or FA.

### BDNF Gene Expression

Analysis of fold change did not reveal differences due to diet in either the right hippocampus or the prefrontal cortex. In the right hippocampus, a BDNF fold change of 1.28 (*P* = 0.156) was observed in the TEST piglets compared with the CONT piglets. Analysis of prefrontal cortex BDNF expression revealed a fold change of 1.27 (*P* = 0.271) in the TEST piglets compared with CONT piglets (data not shown).

## Discussion

In this study, a novel combination of prebiotics, Lf, and MFGM was provided to piglets from 2 to 31 days of age to determine their influence on brain development. We hypothesized that supplementation of prebiotics, Lf, and MFGM would enhance overall brain development in the supplemented group compared with the control group. Previous studies in piglets indicated that supplementation of Lf elicited enhanced spatial learning and memory, as assessed by eight-arm radial maze task (7). Moreover, provision of Lf to young piglets upregulated hippocampal expression of proteins important for learning and memory. Purportedly due to sphingomyelin and phospholipid components, infants supplemented with MFGM exhibited enhanced cognitive development compared with controls, to the point where cognitive scores of MFGM-supplemented subjects were similar to breastfed infants (9). A recent study of preterm infants provided sphingomyelin-fortified milk resulted in faster visual evoked potentials (VEP) at 12 months of age compared with VEP measured at 3 months of age, whereas control infants did not exhibit changes in VEP over this time period. The authors suggested this shorter VEP latency may have been due to increased myelination, resulting from supplementation of sphingomyelin (12). From the present study, MRI outcomes suggested that piglets provided the TEST diet may have had more mature brains when compared with CONT piglets.

Whole brain volumetric analyses indicated no differences due to diet in total brain volume in the present study. Moreover, when anatomical subregions were analyzed relative to total brain volume, there were no observed volumetric differences due to diet. Whereas the vast majority of neurons are established prenatally, growth and expansion of those neurons occurs predominately during the postnatal period (22, 23). Due to prenatal establishment of neuronal numbers, a postnatal dietary supplementation would likely not have a significant impact on whole brain volume, which is consistent with results observed in our study. Moreover, during the postnatal period, the brain experiences the largest relative growth in the cortex, and research suggests piglet cortical growth has not yet reached its maximal growth rate at 4 weeks of age, therefore supporting the lack of difference due to diet until after this time-point (24).

Voxel-based morphometry (VBM) revealed localized differences in gray and white matter tissue concentrations between treatment groups. The most notable differences in gray matter concentrations were observed with CONT piglets exhibiting more and larger clusters of cortical gray matter compared with TEST piglets. While VBM cannot pinpoint the physiological implications of these findings, this decrease in gray matter observed in the TEST group may indicate enhanced neurodevelopment due to this diet. Early in development, an overproduction of synaptic connections occurs, followed by pruning to ensure only necessary neuronal connections are retained (25, 26). Because sialic acid and phospholipids facilitate synapse formation in the developing brain, and these components are found within Lf and MFGM, we speculate that TEST piglets experienced synapse formation followed by pruning earlier than CONT piglets (27, 28). Thus, higher concentrations of gray matter in CONT piglets may indicate regions in which pruning has not yet occurred. To our knowledge, little research has been performed to elucidate the impacts of nutrition on early synaptic pruning, so further research may be warranted in this area. To corroborate these findings, future work is warranted to elucidate the impact of supplemented compounds on cortical tissue, specifically early-developing motor and sensory areas.

Although the exact functional location of motor and sensory areas have not been defined in the piglet, visual inspection of gray matter VBM clusters indicates the observed differences may be in areas related to motor and sensory function (see Figure 2). This conclusion was drawn from locations of human motor and sensory cortices which are centered anterior and posterior to the central sulcus, respectively. A review by Tau and Peterson suggests pruning of motor cortices occurs early in the postnatal period, which serves to corroborate the cortical tissue concentration differences observed in our VBM outcomes (29). Moreover, analysis of white matter concentration was consistent with gray matter, where TEST piglets exhibited localized decreases in concentration of cortical white matter compared with CONT piglets. Assuming that pruning was occurring, it is possible that white matter concentrations would initially decrease due to the reorganization of white matter in affected regions. Taken together, these data suggest that TEST piglets may have been more developmentally advanced compared with CONT piglets. One limitation to this speculation is that the exact timing of pruning and subsequent myelination in the piglet remains unknown. However, our observations were consistent with studies suggesting that cortical regions are rapidly maturing at this age in the piglet (24). A previous study involving piglets supplemented with PL-20 (30), described as a phospholipid-rich milk protein concentrate, showed higher gray and white matter tissue concentrations in supplemented animals. However, due to stark differences in piglet bodyweights, dietary formulations, and feeding regimens, this study does not lend itself to direct comparison with the present results.

In addition to the cortical differences in gray and white matter tissue concentrations shown in VBM, diffusion tensor measures revealed alterations in subcortical tissue microstructure between diets. Analysis revealed differences of RD and MD in the internal capsule between TEST and CONT groups. Piglets provided the TEST diet exhibited lower RD and MD values in the internal capsule compared with CONT piglets. RD is a measure of water movement perpendicular to axon bundles. As myelin maturation occurs around axons, RD tends to decrease due to the restriction of water movement across the axon. MD is a measure of overall water movement in a region, and decreases with age as microstructure increases (31). Thus, lowered RD and MD values observed in our TEST piglets provides further support that piglets on this diet may have experienced enhanced brain development compared with CONT piglets at 30 days of age. Importantly, the internal capsule contains motor and sensory projections from the cortex to corticospinal tract, and is one of the earliest maturing brain regions in the neonatal brain (32). Sphingomyelin is a major component of the MFGM ingredient and, as an integral component of the myelin sheath, supplementation of this compound has previously been speculated to increase myelination in preterm infants (12). Because the measures of RD and MD suggest enhanced subcortical white matter development in the TEST group, it is appropriate to infer that supplementation of the MFGM may have played a role in this observed effect. Thus, based on our observation of possibly enhanced maturation in the internal capsule of piglets, and our assumption that pruning is occurring in sensory/motor areas, our VBM and DTI data provide parallel support for postnatal influence of diet on brain development.

A study of Lf supplementation in piglets revealed increased BDNF expression in the hippocampus and enhanced performance in an 8-arm radial maze task compared with control piglets (7). However, in our study, there were no observed differences in BDNF expression in hippocampal or prefrontal cortical samples due to dietary treatment. Moreover, spatial T-maze behavioral assessment did not yield differences due to diet as assessed by proportion of correct choices. A difference between diets was observed on day 2 of the acquisition phase and days 3 and 4 of the reversal phase in latency to choice. In all instances, piglets in the CONT group were faster at making a choice, whether correct or incorrect, compared with TEST piglets. Latency to choice interpretations tend to vary between behavioral assessments. Some research has implied that shortened latency indicates anxiety-like behaviors, while other research has suggested that shortened latency indicates premature choice or impulsivity (33, 34). While supplemental measures of anxiety and impulsivity were not assessed, our data were consistent with a previous study that indicated administration of bovine Lf in rats resulted in more cautious and less impulsive behaviors (35). As such, we suggest that the presence of Lf in the TEST diet may have attributed to increased latency, and therefore decreased impulsivity, but definitive measures of impulsivity and anxiety are needed to corroborate this evidence.

Considering differences in gene expression and behavioral outcomes from our study were not consistent with those from Chen et al. (7), we speculate this may be due to the differential timing of tissue collection and behavioral assessment between these studies. In our study, participation in the spatial T-maze task started approximately 1 week earlier than the 8-arm radial maze assessment used by Chen et al. (7). Additionally, these authors collected hippocampal tissue at 39 days of age, whereas this time-point was 31 days of age in our study. Based on evidence that developmental trajectory of the piglet brain is nearly identical to that of the human infant, it is suggested that 1 week of piglet brain growth is equivalent to 1 month of human development (24, 36). Thus, 1 week difference in the timing of tissue collection may not allow direct comparisons to be made between studies, simply because piglets may be experiencing different stages of brain development; similar logic may also apply to differential BDNF expression between studies. We propose that future studies using similar dietary treatments would benefit from MRI, behavioral, and tissue assessments at multiple time points, possibly resulting in stronger observed differences between diets. It should be noted that in addition to Lf, MFGM, DHA, ARA, and PDX/GOS were included in the TEST formula in our study, whereas research by Chen et al. (7) was strictly focused on Lf supplementation. More research into the mechanism(s) whereby these compounds interact in development is warranted to elucidate their combined biological effects.

To our knowledge, this is the first study to investigate how a novel combination of prebiotics, Lf, and MFGM influences early postnatal brain development. As such, we suggest that the presence of these dietary ingredients may have elicited enhanced brain development at 4 weeks of age. Neuroimaging outcomes including VBM differences in gray and white matter suggested that TEST-fed piglets experienced axonal pruning earlier than CONT-fed piglets. Moreover, diffusion tensor measures suggested enhanced maturation of the internal capsule, further supporting our hypothesis of increased maturation in TEST piglets compared with CONT piglets. While behavioral assessment did not indicate differences in learning, it is possible that the TEST diet may have reduced impulsivity and/or anxiety, yet further studies are needed to confirm this result. From this study, we conclude that combined dietary supplementation of prebiotics, Lf, and MFGM were well tolerated, supported normal growth (Berding et al., under review)^1^, and positively influenced postnatal brain development in the piglet beyond what is afforded by DHA and ARA.

## Author Contributions

RD, SD, RW, and BB were involved in project conceptualization. AM, LA, and RD were involved in daily project activities. AM, LA, and KB were involved in data collection. AM, LA, RD, KB, and SD were involved in data analysis. All authors were involved in data interpretation and manuscript preparation.

## Conflict of Interest Statement

Sharon M. Donovan and Ryan N. Dilger have received grant funding. Sharon M. Donovan has served on advisory boards. Sharon M. Donovan, Ryan N. Dilger, and Lindsey S. Alexander have consulted for Mead Johnson Nutrition. Brian M. Berg and Rosaline V. Waworuntu are employees of Mead Johnson Nutrition. Austin T. Mudd and Kirsten Berding have no conflict of interest to declare.

## Footnotes

^1^Berding K, Wang M, Monaco MH, Alexander LS, Mudd AT, Chichlowski M, et al. Prebiotics and bioactive milk fractions influence gut development, microbiota and neurotransmitter expression in formula-fed piglets. J Pediatr Gastroenterol Nutr (under review).

## Abbreviations

CONT, control diet; Lf, lactoferrin; MD, mean diffusivity; MFGM, milk fat globule membrane; RD, radial diffusivity; TEST, test diet; VBM, voxel-based morphometry.
